# Hospital tests and patient related factors influencing time-to-theatre in 1000 cases of suspected appendicitis: a cohort study

**DOI:** 10.1186/1749-7922-10-6

**Published:** 2015-01-29

**Authors:** Suzanne Beecher, Donal Peter O’Leary, Ray McLaughlin

**Affiliations:** Department of Surgery, Galway University Hospital/National University of Ireland (NUI), Galway, Republic of Ireland

**Keywords:** Appendicitis, Delay, Time, Factors, Appendicectomy, Appendectomy, Outcome, Readmission, Complications

## Abstract

**Background:**

Acute appendicitis is increasingly being managed in the setting of a dedicated emergency theatre. However understanding of hospital factors that influence time-to-theatre (TTT) is poor. Thus, the aim of this study is to identify factors that influence TTT and to observe the effect of prolonged TTT on patient outcome.

**Methods:**

A retrospective review of an electronic prospectively maintained database was performed over a 2 year period. Factors thought to influence TTT were highlighted. A delay was defined as TTT >8 hours. Data analysis was performed using SPSS 20.

**Results:**

1,000 cases of suspected acute appendicitis were identified. Median age was 19 years. Appendicectomy was performed in 90.7%. 68.1% underwent laparoscopic appendicectomy. Overall mean TTT was 12 hours, 27 minutes. There was a significant association between delayed TTT and female gender (p = 0.017), older age (p = 0.001), pre-operative radiology (<0.001), normal WCC (p < 0.001), normal neutrophils (p < 0.001) and histological non-perforated appendix (p < 0.001). However, on multivariate analysis, younger age, a neutrophilia and presence of a perforation had a shorter TTT. Delayed TTT did not affect outcome variables including post-operative collection (3.59% v 4.38%, p = 0.528), readmission rate (6.54% v 5.72%, p = 0.403) and length of stay (3.1 days v 3.34 days, p = 0.823).

**Conclusions:**

This study highlights key hospital factors that influence TTT in patients with suspected appendicitis. Identification of these influential factors adds greatly to our understanding of patient prioritisation. Finally, TTT delays greater than 8 hour do not appear to affect short-term patient outcomes.

## Introduction

Acute appendicitis is recognised internationally as the most common acute surgical emergency requiring surgical intervention [1]. Acute appendicitis is increasingly being managed in the setting of a dedicated emergency theatre [2, 3]. Provision of dedicated emergency theatre facilities has resulted in improved patient outcomes including a shorter length of stay and improved morbidity and mortality rates [4, 5].

There is much debate surrounding the timing of appendicectomy performance at present [6]. In order to avoid out of hours appendicectomies being performed, delays may be encountered [7]. Recent published data has noted that delays up to 24 hours do not appear to increase the complexity of appendicitis or associated morbidity [8, 9]. On this basis it appears delays less than 24 hours may not adversely affect patient outcome, however timely intervention is still warranted in specific groups for control of sepsis and pain [10, 11].

Provision of timely intervention is very much dependent upon an efficient emergency theatre [12]. Despite having dedicated emergency theatres, delays are still encountered on a daily basis between the time when a decision is made to go to theatre and the time of intervention with costly implications [13]. A lack of capacity to facilitate emergency cases for all surgical specialties presumably is the main factor contributing to delays however there is a paucity of information within the literature pertaining to the factors that influence the time to theatre (TTT) in patients with suspected acute appendicitis. Identification of these factors would greatly enhance our understanding of TTT in suspected acute appendicitis and aid the organisation of emergency surgery services.

Thus we wished to identify the hospital and patient related factors that influence TTT in patients with suspected appendicitis, paying particular attention to patient, procedure and histological factors. We then wished to examine the effect of delayed TTT on short term patient outcomes following appendicectomy.

## Methods

An electronic prospectively maintained emergency theatre database was used to identify patients with suspected appendicitis between 31st December 2011 to the 31st December 2013.

Data collected included patient gender, patient age, patient identification number, operation, time added to list, operation start time and operation finish time. We also noted the surgical approach used, either laparoscopic or open. Blood parameters were recorded including White Cell Count (WCC), neutrophils and C-reactive protein (CRP). Use of additional radiological diagnostic modalities pre-operatively was recorded. Outcome variables including length of stay, post-operative collection, conversion to open, readmission and perforation rates were recorded. We did not gather data relating to wound infections as outpatient management of these complications would have provided incomplete and inaccurate data.

The time at which the patient was added to the emergency theatre list represented the time that the decision was made to go to theatre at the time of surgical admission in the Emergency Depatment. A delayed time to theatre for the operative management of acute appendicitis of >8 hours was defined by priority targets for emergency theatre access in emergency surgery guidelines and publications. We thus defined a delayed appendicectomy as >8 hours following the decision to intervene. Out of hours was defined as an operation start time outside the hours of 9 am to 5 pm.

A univariate analysis was performed to identify factors associated with a delayed TTT. Significant factors were then entered into a multivariate analysis and analysed using a logistic regression model to identify independent factors which influenced TTT. Statistical analysis was performed using SPSS version 20.0. A p-value <0.05 was considered statistically significant. Ethics approval was received for the ethics committee at University Hospital Galway.

## Results

1000 cases of suspected acute appendicitis were identified from our prospectively maintained emergency theatre electronic database and we subsequently performed a detailed analysis of these cases.

Patient demographics and operative details are outlined in Table 1. The median patient age was 19 years. Of the 1000 patients who had clinical evidence of appendicitis, appendicectomies were performed in 90.7% of patients, with the majority of patients (68.1%) undergoing laparoscopic appendicectomy. In patients who were <12 years, open appendicectomy was more frequently performed (62.5%).

**Table 1:** **Patient demographics & operative details**

Total		N = 1000	
**Demographics**			
	Age* (range)	19 (2–78)	
	Males	435	
	Females	565	
	Total Patients ≤12 years	264	
	Females ≥16 years	360	
	Pregnant Patients	4	
**Operative details**			
	Laparoscopy Only	93	
	Appendicectomy	907 90.7%	
	Laparoscopic Appendicectomy	618	68.1%
	Open Appendicectomy	228	25.1%
	Conversion to Open	18	2%
	Right Hemicolectomy	3	0.33%
	Unknown	40	4.4%

*Median.

Of the 907 appendicectomies performed, pathology was detected in 730 cases, providing a negative appendicectomy rate of 19.6%. Table 2 details the histopathology findings on the resected appendixes. Acute appendicitis was found in 65.5% specimens. 11.8% of patients had a perforated appendix. A carcinoid tumour was detected in 1%.

**Table 2:** **Histopathology from appendicectomies performed**

Appendicectomies			N = 907
Pathology			730
	Acute appendicitis	603	
	Suppurative/Gangrenous	107	
	Enterobius Vermicularis	323	
	Carcinoid	9	
	Faecolith	199	
	Faecolith + appendicitis	93	
	Faecolith – appendicitis	106	
No pathology			177

We next looked at factors influencing TTT, as highlighted by Table 3. The overall mean time to theatre was 12 hours, 27 minutes. A number of factors were found to be significant on univariate analysis. Younger patients were brought to theatre quicker than older patients (p = 0.001). Male patients endured a shorter time awaiting surgery (p = 0.017). Patients with abnormal blood parameters, particularly those with a raised neutrophil count on presentation, were also brought to theatre faster than those with normal parameters (p < 0.001). Those operated on within 8 hours were more likely to have their appendix removed (P = 0.035). Retrospectively, those with histopathological confirmation of a perforated appendix had also been brought to theatre more rapidly than those without (p < 0.001). Pre-operative radiology increased TTT by 52% in those patients who underwent either an ultrasound or CT scan (p < 0.001).

**Table 3:** **Factors influencing TTT**

	<8 Hours (447)	≥8 Hours (553)	Univariate p-value	Multivariate p-value
**Patient age (mean)**	20.6	23.4	0.001	0.027
**Gender (N)**				
**Male**	212	223		
**Female**	235	330	0.017	0.552
**Radiology Pre-op (N)**	81	180	<0.001	0.836
**Ultrasound**	60	148	<0.001	0.192
**Abnormal bloods (N)**				
**WCC**	304	284	<0.001	0.750
**Neutrophils**	311	284	<0.001	0.022
**CRP**	290	297	<0.001	0.160
**Operation type (N)**				
**Diagnostic laparoscopy**	32	61		
**Appendicectomy**	413	488	0.035	0.824
**Perforation (N)**				
**Yes**	66	41		
**No**	349	451	<0.001	0.01

On multivariate analysis, younger age, a raised neutrophil count and the presence of a perforation were found to be independent factors influencing TTT.

Table 4 displays the impact of theatre delay in those who had histologically confirmed appendicitis. Interestingly, there was no significant difference between any of the outcome variables examined with or without an 8 hour delay.

**Table 4:** **Impact of theatre delays on patient outcomes for those with appendicitis**

	Theatre <8 hrs (N = 306)	Theatre ≥8 hrs (N = 297)	***P***-value
**Perforation**	66 (21.5%)	41 (13.8%)	0.008
**Conversion to open**	6 (1.96%)	11 (3.7%)	0.148
LOS* ( ***range*** **)**	3 days (1–12)	3 days (1–20)	0.488
**Readmission**	20 (6.54%)	17 (5.72%)	0.403
**Collection**	11(3.59%)	13 (4.38%)	0.528

*Median.

Table 5 compares outcomes of those operated on during working hours and those operated on out of hours. Time of the day did not have a significant impact on TTT. There was no significant difference in the outcomes for appendicectomies performed out of hours.

**Table 5:** **Subanalysis of appendicectomies performed out of hours**

	Working hours	Out of hours	P value
Time to theatre*	10:43	14:23	0.215
Length of stay*	3 days	3 days	0.481
**Readmission (N)**	29	33	0.804
**Morbidity (N)**	30	39	0.406

*Mean, hh:mm.

## Discussion

Organisation of emergency surgery services requires a greater understanding of the factors influencing how we prioritise use of limited resources within the emergency setting. This study identifies three key factors which influence the TTT for patients with suspected appendicitis. These patient related factors provide useful parameters on which to prioritise patients with suspected appendicitis appropriately for emergency theatre.

Understandably, younger aged patients were identified as having quicker access to theatre. Our emergency theatre prioritisation system ensures children <12 years of age in need of an emergency operation are given high priority and invariably have their intervention in a timely manner. This is to the detriment of older patients but is necessary in the setting of limited theatre resources. Interestingly, a neutrophilia was found to be the only blood parameter found to influence TTT. A neutrophilia indicates an acute inflammatory response and would be in keeping with an acute appendicitis and appears to represent a good marker of the clinical severity of the appendicitis and the timing of appendicectomy. Histological confirmation of a perforated appendix was the final factor that influenced TTT. Although presence of a perforation is a factor that is determined retrospectively, these patients would have more advanced clinical signs and signs of sepsis. This indicates that clinical exam is indispensible in deciding who should be operated on urgently. Altogether, these patient related factors highlight the factors that influence our decision making when prioritising patients for an appendicectomy in the setting of a multi-speciality emergency theatre. It is clear that clinical prioritisation influences TTT with young age, increased neutrophils and presumably clinical signs suggesting perforation resulting in the surgeons being more proactive in prioritising these cases. It is also clear that while there were no clear increased complications in the group with longer TTT it is important to realise that this is not the same as saying all patient can wait. The lack of increased complication rate in the delayed group may be as a result of sicker patients being prioritised to earlier theatre times.

This study highlights the patient related factors which influenced TTT. It is likely there are other non-patient related organisational factors which also have a bearing on TTT such as staffing levels for example. However these would difficult to measure and would vary from institution to institution. The lack of influence of pre-operative radiology on TTT was surprising. However, the decision for theatre would not have been taken until after the radiological investigation, thus although the overall length of stay was delayed, the TTT by definition was not. Interestingly the time of day does not impact on TTT.

Delaying TTT beyond the 8 hour time frame does not appear to affect the short-term outcome for patients with acute appendicitis within this study. Although this time-frame was defined by published guidelines and recent studies, we also analysed this data looking at different time-points including 8,12,16 and 24 hours [2, 12]. There was no difference noted in patient short-term outcome at any of these time-points. This is consistent with findings from a recent meta-analysis and systematic review which demonstrated delays up to 24 hours in selected patients may be feasible as studies have shown that a delay within this window is not associated with increased rates of complex pathology [6]. Thus there may be scope to extend the recommended TTT within current emergency surgery guidelines, however those with clinical signs of perforation, neutrophillia and younger age were prioritised in this study which may suggest the subgroups that need early surgery.

## Conclusion

This study highlights key ‘patient related’ factors that influence TTT in patients with suspected appendicitis. Identification of these influential factors adds greatly to our understanding of patient prioritisation in the setting of a dedicated emergency theatre. Finally, TTT delays greater than 8 hour do not appear to affect short-term patient outcomes.
